# EMERGING TECHNOLOGY-BASED INTERVENTIONS TO SUPPORT FAMILY CAREGIVERS

**DOI:** 10.1093/geroni/igad104.1751

**Published:** 2023-12-21

**Authors:** Tiffany Washington, Nicole Ruggiano, Joseph Gaugler

## Abstract

There are 53 million family caregivers in the U.S., most of whom experience difficulty coordinating care, health and mental health decline, social isolation, and loneliness. Furthermore, accessing community support services remains challenging. This gap in access can create a significant threat to caregivers’ wellbeing and quality of life. The need to develop innovative interventions to support family caregivers is urgent. This symposium will describe the potential of leveraging emerging technology-based interventions to support family caregivers. Speaker one will outline considerations for design and development of social engagement interventions for caregivers delivered via video-conferencing platforms. Speaker two will describe the development and initial testing of a touchscreen mobile application that supports communication between people living with dementia and their caregivers to promote person-centered care. Speaker three will describe the human-centered design of a digital health solution to improve symptom management in advanced cancer care for older adults, emphasizing specific strategies to incorporate evidence-based principles of digital inclusivity. Speaker four will present findings from a proof of concept study examining the acceptability of a virtual respite program for caregivers of individuals with dementia. Speaker five will explore the potential for crowdsourced mapping technologies to support caregivers of people living with dementia. The discussant will tie together themes, offer recommendations for future intervention research with family caregivers, and discuss implications of emerging technologies on practice, policy, and research.

